# Sleep phenotype in the Townes mouse model of sickle cell disease

**DOI:** 10.1007/s11325-018-1711-x

**Published:** 2018-08-29

**Authors:** Brett J. O’Donnell, Lanping Guo, Samit Ghosh, Faraaz A. Shah, Patrick J. Strollo, Bryan J. McVerry, Mark T. Gladwin, Solomon F. Ofori-Acquah, Gregory J. Kato, Christopher P. O’Donnell

**Affiliations:** 1 Division of Pulmonary, Allergy and Critical Care Medicine, University of Pittsburgh School of Medicine, 3459 Fifth Avenue, MUH 628 NW, Pittsburgh, PA 15213 USA; 2 Division of Hematology/Oncology, Department of Medicine, University of Pittsburgh School of Medicine, 3459 Fifth Avenue, MUH 628 NW, Pittsburgh, PA 15213 USA

**Keywords:** Sickle cell disease, Sleep, Arousals, Hemoglobin

## Abstract

**Purpose:**

Patients with sickle cell disease (SCD) regularly experience abnormal sleep, characterized by frequent arousals and reduced total sleep time. However, obstructive sleep apnea syndrome (OSAS) is a common comorbidity of SCD, making it unclear whether the disease per se is impacting sleep, or sleep disruption is secondary to the presence of OSAS. Thus, we assessed sleep, independent of OSAS, using a mouse model of SCD.

**Methods:**

Sleep was compared between 10-to-12-week-old Townes knockout-transgenic mice with the sickle cell phenotype SS (*n* = 6) and Townes mice with sickle cell trait AS (*n* = 6; control). The mice underwent chronic polysomnographic electrode implantation (4EEG/2EMG) to assess sleep architecture.

**Results:**

The SS mice had significantly lower hemoglobin concentration compared to control AS mice (7.3 ± 1.3 vs. 12.9 ± 1.7 g/dL; *p* < 0.01), consistent with the expected SCD phenotype. SS mice exhibited significantly decreased total NREM sleep time (45.0 ± 0.7 vs. 53.0 ± 1.3% 24 h sleep time; *p* < 0.01), but no change in total REM sleep time compared to the AS mice. The SS mice took longer to resume sleep after a wake period compared to the AS mice (3.2 ± 0.3 min vs. 1.9 ± 0.2 min; *p* < 0.05). Unexpectedly, SS mice experienced fewer arousals compared to AS mice (19.0 ± 0.9 vs. 23.3 ± 2.1 arousals/h of sleep; *p* = 0.031).

**Conclusions:**

The presence of decreased total NREM sleep associated with reduced arousals, in the absence of OSAS, suggests a distinctive underlying sleep phenotype in a mouse model of SCD.

## Introduction

Sickle cell disease (SCD) is one of the most prevalent hemoglobinopathies in the USA with roughly 1 in 350 newborns in the African-American population affected [1]. SCD is a genetic disease of the red blood cell in which deoxygenation produces an abnormal sickle shape of the cell, leading to vaso-occlusion, tissue ischemia, and infarction, as well as chronic hemolytic anemia [2]. Advancements in the understanding of SCD have resulted in better management and treatment of cardiovascular complications associated with the disease [3], but many other complications of SCD remain both poorly defined and often untreated.

Sleep abnormalities, as a potential complication of SCD, remain poorly defined. It is known that pediatric SCD patients experience an increased occurrence of obstructive sleep apnea syndrome (OSAS), with prevalence rates of OSAS ranging from 10 to 79%, compared to rates of 1–5% in the general population [4]. OSAS can cause significant disruptions in sleep architecture and may account for the increased arousal frequency and reduced total sleep time reported in pediatric SCD patients [5]. Indeed, children with SCD undergoing adenotonsillectomy exhibited improved total sleep time and fewer arousals [6]. The use of animal models allows the impact of the SCD genotype on sleep architecture to be studied independent of OSAS, or other significant comorbidities.

We, therefore, chose to study the impact of the SCD genotype on sleep in mice, since upper airway stability precludes the development of OSAS, even in obese animals [7]. The Townes SS mouse, which contains two human sickle hemoglobin alleles, exhibits the classic hematologic sickle phenotype with sickle-shaped erythrocytes, increased reticulocytes, and decreased hemoglobin concentration [8, 9]. The Townes AS mouse, which has one human sickle allele and one human adult hemoglobin allele, exhibits little to no symptoms of SCD, providing a rigorous control relative to the Townes SS mouse. We hypothesized that if the SCD phenotype has any effect on sleep pathology independent of OSAS, then SS mice would exhibit impaired sleep architecture compared to AS control mice.

## Methods

### Animals

Experiments were conducted in adult male 10-to-12-week-old Townes knockout-transgenic SS and AS sickle cell mice on a background strain of B6 and S129. Colonies of Townes SS and AS mice were bred and maintained at the University of Pittsburgh, as previously reported [10]. Mouse genotypes were confirmed either by PCR or Hb gel electrophoresis at 3–4 weeks of age. Mouse phenotypes were confirmed by total Hb concentration (from venous whole blood using a portable Co-oximeter; AVOXImeter 4000) and reticulocyte count (from flow cytometry) at 10 weeks of age. Animal handling and experimentation was conducted ethically and in accordance with approved Institutional Animal Care and Use Committee (IACUC) protocols at the University of Pittsburgh.

### Protocol

All mice underwent surgical implantation of polysomnographic electrodes on day 0 and were tethered to the recording system after 7 days of recovery. On day 11, after 4 days of undisturbed acclimation to the tether, electroencephalographic (EEG) and electromyographic (EMG) signals were recorded for a continuous 24-h period. A subset of mice were subsequently catheterized on day 12, and, after 2 days of recovery, arterial blood was collected under freely behaving conditions for assessment of arterial blood gases.

### Surgical instrumentation

Animals were anesthetized using 1–2% isoflurane for all surgical procedures, administered pain medication after surgery (0.3 mg/ml buprenorphine for maximum of 3 days), and monitored daily during the post-operative period. EEG electrodes (E363/1, Plastics One, Roanoke, VA) and nuchal EMG electrodes (E363/76, Plastics One) were implanted as previously described [11]. In brief, a midline incision was made to expose the skull and muscles immediately posterior to the skull. The underlying fascia was gently cleared from the skull surface, four small burr holes were drilled through the skull in the frontal and parietal regions, and four EEG electrodes were fastened via jewel screws (diameter of 1.6 mm). The first and second electrodes were placed 2–3 mm caudal to bregma and 1–2 mm lateral of the midsagittal suture. The third and fourth electrodes were placed 2–3 mm rostral to bregma and 1–2 mm lateral of the midsagittal suture. Two nuchal EMG electrodes were stitched flat onto the surface of the neck muscle. The EEG and EMG electrodes were inserted into a pedestal (MS363, Plastics One) and secured to the skull with dental acrylic. After recovery from surgery, a connector cable was placed onto the head pedestal and attached to a low-friction mercury swivel allowing unrestricted movement of the tethered mouse.

In a subset of mice, an arterial catheter was implanted chronically under isoflurane anesthesia, as previously described [12]. The catheter was inserted in the left femoral artery, sutured in place, stabilized with superglue (Henkel Corp., Rocky Hill, CT, USA), and tunneled subcutaneously to the upper back by threading through a blunt needle. The catheter was taped to the connector cable attached to the pedestal and connected to a 360° swivel designed for mice (375/D/22QM; Instech, Plymouth Meeting, PA, USA) that worked in combination with the electrical swivel used to record polysomnography. Catheter patency was maintained by continuously flushing saline containing 20 U ml^−1^ heparin (Baxter, Deerfield, IL, USA) at a rate of 3 μl h^−1^ using a multi-syringe pump (R99-EM; Razel Scientific Instruments, St. Albans, VT, USA).

### Housing

The mice were maintained on a 12:12-h light-dark cycle and were housed in a customized pyramidal cage [7″ (W) × 9″ (H) × 7″ (L)] with continuous access to food and water. The cage was contained inside a light-controlled and sound-dampening chamber [22″ (L) × 16.5″ (H) × 14″ (W)] (BRS/LVE, Laurel, MD). The mice were housed in the same chambers throughout the entire adaptation and experimental period to control for environmental exposure. The polysomnographic tether and arterial catheter (in the subset of instrumented mice) exited through a 1″-diameter hole in the top of the chamber, and connected to an amplifier and a multi-syringe pump, respectively.

### Data acquisition

A Grass Instruments amplifier (Quincy, MA) was used to record EEG activity (filtered 0.1–30 Hz) and EMG activity (filtered 10–100 Hz). Signals from the Grass recorder were collected using Windaq Pro acquisition software (Dataq Instruments; Akron, OH), were digitized at 300 Hz (DI-720 data acquisition board; Dataq Instruments; Akron, OH), and stored for analyses.

### Arterial blood gas analyses

Approximately 80 μl of arterial blood was collected and analyzed with a VetScan i-STAT 1 blood gas analyzer (ABAXIS, Union City, CA). Blood samples were withdrawn during the light period with the mouse in quiet wakefulness to determine the partial pressure of arterial oxygen (PaO_2_), the partial pressure of arterial carbon dioxide (PaCO_2_), and the arterial oxygen saturation (SO_2_).

### Sleep scoring

Sleep data were analyzed using a customized program that converted DATAQ digitized data files into Stanford Sleep Structure Scoring System (SSSSS) format for characterization of signals using the rodent software developed by Joel H. Benington [13] and subsequently validated in mice by Veasey et al. [14]. The program utilizes Fourier spectral analysis of the EEG in the delta (0.5–4.0 Hz), sigma (10.0–14.0 Hz), and theta (6.0–9.0 Hz) frequency bands in combination with the moving average of the EMG amplitude to assess sleep/wake states in 10-s epochs (necessary to provide accurate determination of the beginning and end of REM sleep bouts that typically average around 1 min). Twenty-four-hour periods of data were plotted as sigma*theta power from Fourier spectral analysis against EMG, and thresholds for the slope and intercept of the relationship were used to distinguish between sleep and wake. A second plot of the delta/theta power from Fourier spectral analysis against EMG was used to distinguish non-rapid eye movement (NREM) sleep from rapid eye movement (REM) sleep based on a delta/theta threshold.

Total time spent in each of the three sleep states (Awake, NREM, and REM) was calculated as a percentage of the number of epochs of that state over a 24-h period. The number and duration of NREM and REM sleep bouts were determined according to the following criteria: an NREM sleep bout began with three or more consecutive epochs of NREM sleep and ended with three or more consecutive epochs of wake or two or more consecutive epochs of REM sleep; an REM sleep bout started with two or more consecutive epochs of REM sleep and ended with three or more consecutive epochs of wake or NREM sleep. An awake bout began with three consecutive epochs of awake and ended with three consecutive epochs of sleep (any combination of NREM or REM sleep). Time to resume sleep was defined as the length of time from the onset of three or more consecutive epochs of wake to the next combination of any three consecutive epochs of NREM or REM sleep. An arousal was defined as one or more epochs of wake following three or more consecutive periods of either REM or NREM sleep. Arousal frequency per hour of sleep was calculated as total number of arousals/total sleep time in 24 h.

### Statistics

All results are presented as means ± standard error of the mean (SEM). Statistical differences between groups were determined by two-way unpaired Student’s *t* test.

## Results

### Blood parameters

The AS and SS mice were matched for age and weight and arterial blood gas analyses showed no difference in PaO_2_, PaCO_2_, SO_2_, or pH between strains (Table 1). As expected, the SS mice exhibited significantly less (*p* < 0.01) total hemoglobin compared to AS mice, as well as a significantly higher (*p* < 0.01) reticulocyte percentage of red blood cells (Table 1).

**Table 1:** Age, weight, and arterial blood gas profiles for Townes AS and SS mice

	AS (*n* = 6)	SS (*n* = 6)	*p* value
Age (weeks)	11.4 ± 0.9	11.8 ± 0.5	= 0.72
Body weight (gm)	28.9 ± 1.4	29.8 ± 1.4	= 0.60
Total hemoglobin (g/dL)	12.9 ± 1.7	7.3 ± 1.3	< 0.01
Reticulocyte (% RBCs)	9.2 ± 3.8	57.8 ± 6.1	< 0.01
PaO_2_ (mmHg)	93 ± 2	92 ± 1	^a^
PaCO_2_ (mmHg)	38.8 ± 0.8	38.2 ± 1	^a^
SO_2_ (% Hb)	97.3 ± 0.5	96.2 ± 1	^a^
pH	7.44 ± 0.051	7.38 ± 0.071	^a^

Data shown as mean ± SEM. Partial pressure of arterial oxygen (PaO_2_), partial pressure of arterial carbon dioxide (PaCO_2_), and arterial oxygen saturation (SO_2_). Statistical differences determined by two-tailed unpaired Student’s *t* test; ^a^statistics are not included for arterial blood gas data collected due to small sample size (AS: *n* = 3 and SS: *n* = 2)

### Polysomnographic findings

**Fig. 1:**
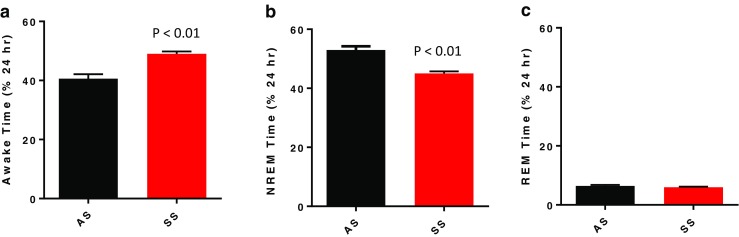
Mean ± SEM for time spent awake (**a**), in non-rapid eye movement sleep (NREM; **b**), and rapid eye movement sleep (REM; **c**) over a 24-h period in Townes mice with the sickle cell phenotype SS (*n* = 6) and Townes mice with the sickle cell trait AS (*n* = 6). Statistical differences determined by two-tailed unpaired Student’s *t* test

**Fig. 2:**
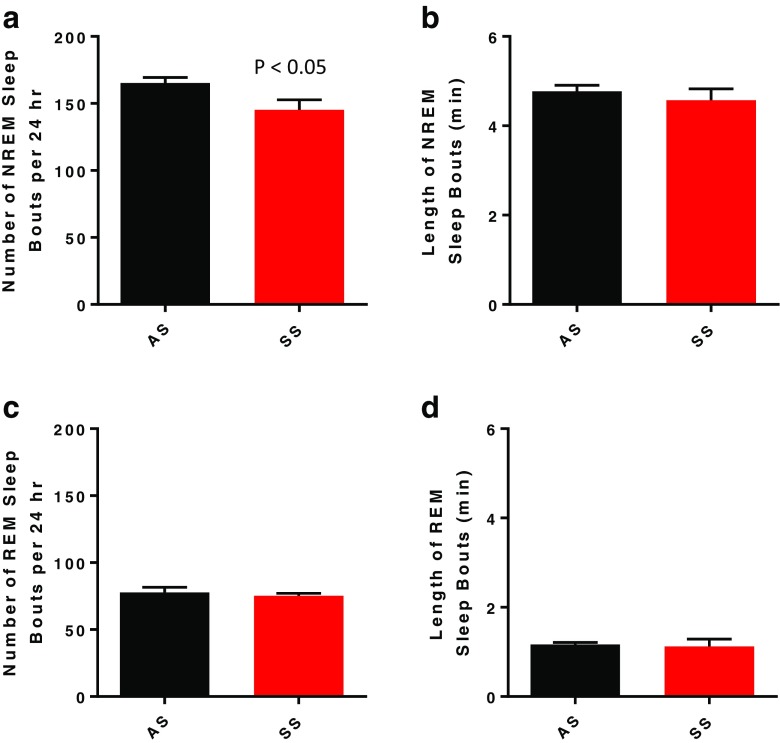
Mean ± SEM for number (**a**) and duration (**c**) of non-rapid eye movement (NREM) sleep bouts and number (**b**) and duration (**d**) of rapid eye movement (REM) sleep bouts over a 24-h period in Townes mice with the sickle cell phenotype SS (*n* = 6) and Townes mice with the sickle cell trait AS (*n* = 6). Statistical differences determined by two-tailed unpaired Student’s *t* test

Over the 24-h sleep/wake cycle, SS mice exhibited a 17% reduction of time in NREM sleep, with a comparable increase in time awake (Fig. 1a, b; *p* < 0.01) relative to AS mice. The decrease in NREM sleep time was due to a decrease in number, but not the duration, of NREM bouts (Figs. 1a and 2b). Interestingly, when assessing just the dark (active) period, the SS mice did exhibit a significant decrease (*p* < 0.05) in NREM bout length compared to the AS mice (Table 2; *p* < 0.05). In contrast to NREM sleep, there were no differences in time spent in REM sleep in either the light or dark period and no differences in number or duration of REM sleep bouts between the SS and AS mice (Figs. 1c and 2c, d and Table 2).

**Table 2:** Distribution of sleep characteristics in 12-h light and dark periods for Townes AS and SS mice

	LIGHT			DARK		
	AS	SS	*p* value	AS	SS	*p* value
Awake (% of 12 h)	35.6 ± 1.8	39.5 ± 1.9	= 0.17	45.6 ± 2.3	58.6 ± 1.2	< 0.01
NREM (% of 12 h)	56.8 ± 1.4	52.8 ± 1.4	= 0.06	49.1 ± 2.0	37.2 ± 0.7	< 0.01
REM (% of 12 h)	7.6 ± 0.5	7.7 ± 0.5	= 0.84	5.3 ± 0.4	4.2 ± 0.4	= 0.19
Number of NREM bouts	93.0 ± 2.9	81.0 ± 4.2	< 0.05	72.2 ± 2.9	64.3 ± 3.5	= 0.12
Duration of bouts (min)	4.5 ± 0.1	4.8 ± 0.3	= 0.45	5.1 ± 0.3	4.3 ± 0.2	< 0.05
Number of REM bouts	47.0 ± 3.3	47.8 ± 2.6	= 0.85	30.7 ± 2.0	27.3 ± 2.8	= 0.36
Duration of bouts (min)	1.1 ± 0.03	1.1 ± 0.02	= 0.94	1.2 ± 0.1	1.0 ± 0.1	= 0.18
Number of arousals/h sleep	23.9 ± 2.7	17.1 ± 1.2	< 0.05	22.9 ± 1.4	19.6 ± 1.3	= 0.13
Time to resume sleep (min)	1.6 ± 0.1	2.4 ± 0.3	< 0.05	2.4 ± 0.3	4.2 ± 0.5	< 0.01

Data shown as mean ± SEM and statistical differences determined by two-tailed unpaired Student’s *t* test

**Fig. 3:**
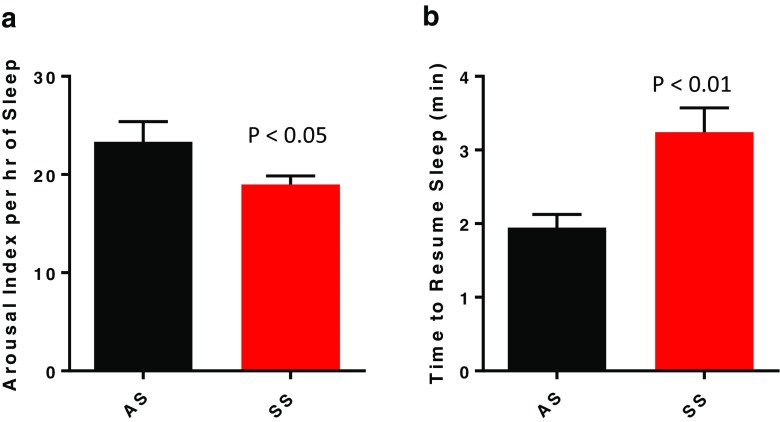
Mean ± SEM for number of arousals per hour of sleep (**a**) and time to resume sleep (**b**) over a 24-h period in Townes mice with the sickle cell phenotype SS (*n* = 6) and Townes mice with the sickle cell trait AS (*n* = 6). Statistical differences determined by two-tailed unpaired Student’s *t* test

Despite the SS mice spending less time in NREM sleep than AS mice, they exhibited significantly fewer arousals per hour of sleep (Fig. 3a; *p* < 0.05). A decrease in arousal frequency in the light period primarily accounted for the overall 24-h decrease in arousal frequency (Table 2; *p* < 0.05). The time to resume sleep after an arousal was significantly increased in SS mice compared to AS mice over the 24-h sleep/wake cycle (Fig. 3b; *p* < 0.01), a response that was present in both the light and dark periods (Table 2).

## Discussion

Little is known about sleep in SCD patients, and what is known is confounded by multiple comorbidities associated with the disease, including a high prevalence of OSAS. Thus, it is unclear whether the decreased total sleep time and fragmented sleep reported in SCD patients [5, 15–17] represents a comorbid response to the presence of OSAS, or if there is a more fundamental defect related to the sickle cell genotype. Studying young and lean Townes mice provides an exciting opportunity to examine the impact of SCD on sleep independent of phenotypic differences in arterial blood gas concentrations and in the absence of OSAS. Our data show a sleep phenotype in SS mice characterized by an increased time to fall asleep and an associated reduction in NREM sleep time, but, surprisingly, once sleep is initiated, it is less fragmented.

To our knowledge, there are no data showing that reduced sleep time in SCD patients is associated with an improved consolidation of sleep. Indeed, objective assessment of sleep in SCD indicates a phenotype of increased arousal frequency and reduced total sleep time [4]. The high prevalence of OSAS in SCD patients would be expected to fragment sleep, and other chronic conditions common in SCD, such as enuresis [18], priapism [19], and periodic leg movement disorder [20], would also be expected to fragment rather than consolidate sleep. Yet, in young Townes mice, the SS genotype infers a distinctive phenotype where NREM sleep is reduced, but paradoxically consolidated.

It is possible that anemia per se in the Townes model may contribute to some of the sleep phenotypic responses observed. For example, a previous study from our laboratory demonstrated that chronic iron deficiency led to a specific, but small, decrement in NREM sleep in the last 4 h of the dark (or active) period, simulating clinical restless leg syndrome in patients [21]. Since the degree of anemia induced by chronic iron deficiency was less than observed in the SS mice used in the current study, we cannot exclude the possibility that greater anemia may contribute to some aspect of the sleep phenotype we report in SS mice. Nevertheless, given there was no change in arousal frequency related to iron deficiency, the distinctive phenotype of reduced NREM sleep associated with a reduced arousal frequency observed in the SS mice is likely dependent on mechanisms beyond anemia alone.

What other aspects of the SCD phenotype could impact sleep architecture? If the SS mice experience chronic cerebral ischemia from vaso-occlusion as reported in SCD patients, there could be a direct effect of reduced blood flow to sleep-controlling regions in the central nervous system. Alternatively, if vaso-occlusion induces pain in SS mice from local tissue hypoxia secondary to obstruction of blood flow, as described in SCD patients [22], sleep could be impacted. In SS mice, the reduction in NREM sleep time and increased time to resume sleep after awakening was most prominent in the dark, or active, period of the mice and potentially could be attributed to pain when awake, disrupting the ability of SS mice to fall asleep and contributing to the reduction in NREM sleep. In the predominant sleep phase during the light period, however, once sleep ensues, the perception of pain would be lessened, potentially decreasing arousal frequency. Recent studies in Townes mice suggest, at least for the lung, that vaso-occlusion does not spontaneously occur in the absence of a lipopolysaccharide challenge [23]. Whether there is direct vaso-occlusion in the central nervous system or vaso-occlusion in peripheral organs other than the lung that could induce a pain response in Townes SS mice remains to be determined.

Another common pathophysiology of SCD is hemolysis, which liberates hemoglobin from intravascularly hemolyzed sickle erythrocytes. Hemoglobin in the plasma can consume nitric oxide inducing an abnormal vasoconstriction [24]. Hemolysis-induced vasoconstriction could function in a similar manner to vaso-occlusion by restricting blood to sleep-controlling regions in the brain. Interestingly, our observation that the reduction in total sleep time in SS mice occurs in NREM, but not REM, sleep suggests that if there is a direct impact of the SS genotype on the central nervous system, then it likely affects specific brain regions or neural pathways controlling these sleep states.

It is important to provide context for our findings of reduced NREM sleep time in the Townes SS mice regarding a phenotype that effectively models sleep deprivation. In a previous study, we experimentally reduced NREM sleep by exposing C57BL/6J mice to either intermittent hypoxia or sleep fragmentation in the light/sleep period and observed a concomitant rebound of REM sleep in the subsequent period of undisturbed sleep in the dark/active period [25]. Given that in the present study REM sleep was comparable between Townes SS and AS mice (Fig. 1c), our data suggest that the reduced NREM sleep we observe in the Townes SS mice is not inducing a characteristic sleep deprivation response. However, given the patterns of sleep we observed in Townes mice, we cannot rule out the possibility of disturbances in circadian rhythm contributing to the SS phenotype. As such, exploring the impact of SCD on circadian rhythm could be an interesting area for future studies in human SCD patients as well as animal models of SCD.

In summary, our data show that a distinctive phenotype of reduced, but more consolidated, sleep exists in SCD mice. A potential implication of our study is that to effectively treat sleep abnormalities in SCD patients and improve their quality of life, addressing only the comorbid presence of OSAS may not be sufficient to normalize sleep in the clinical setting. A two-part treatment approach may be required: first, to treat the comorbidities and related symptoms, and second, to treat the effects of the SCD genotype on sleep.
